# Sphingolipid metabolism and signaling in cardiovascular diseases

**DOI:** 10.3389/fcvm.2022.915961

**Published:** 2022-08-31

**Authors:** Sonia Borodzicz-Jażdżyk, Piotr Jażdżyk, Wojciech Łysik, Agnieszka Cudnoch-Jȩdrzejewska, Katarzyna Czarzasta

**Affiliations:** ^1^Chair and Department of Experimental and Clinical Physiology, Laboratory of Centre for Preclinical Research, Medical University of Warsaw, Warsaw, Poland; ^2^Second Department of Psychiatry, Institute of Psychiatry and Neurology in Warsaw, Warsaw, Poland

**Keywords:** sphingolipids, heart failure, hypertension, coronary artery disease, ceramides, sphingosine-1 phosphate, stroke, cardiovascular disease

## Abstract

Sphingolipids are a structural component of the cell membrane, derived from sphingosine, an amino alcohol. Its sphingoid base undergoes various types of enzymatic transformations that lead to the formation of biologically active compounds, which play a crucial role in the essential pathways of cellular signaling, proliferation, maturation, and death. The constantly growing number of experimental and clinical studies emphasizes the pivotal role of sphingolipids in the pathophysiology of cardiovascular diseases, including, in particular, ischemic heart disease, hypertension, heart failure, and stroke. It has also been proven that altering the sphingolipid metabolism has cardioprotective properties in cardiac pathologies, including myocardial infarction. Recent studies suggest that selected sphingolipids may serve as valuable biomarkers useful in the prognosis of cardiovascular disorders in clinical practice. This review aims to provide an overview of the current knowledge of sphingolipid metabolism and signaling in cardiovascular diseases.

## Introduction

Sphingolipids are a structural component of the cell membrane, including cardiomyocytes, neurones, and microglia among others (1, 2). Sphingolipids are derived from sphingosine, which owes its name to its unusual structure, placing it between the amines and alcohols (3). The sphingoid base of sphingosine undergoes various types of enzymatic transformations and this leads to the formation of biologically active compounds, which play a crucial role in the essential pathways of cellular signaling, proliferation, maturation, and death (4). The most important representatives of sphingolipids are ceramide (Cer) and sphingosine (Sph), Sph-1-phosphate (S1P), and Cer-1-phosphate (C1P) (5). It should be noted that Cer can be formed by *de novo* synthesis, but it can also be formed as a result of the catabolism of complex sphingolipids. Sphingolipids are degraded in lysosomes by the endosomal/lysosomal membrane digestion system (6). Disorders of individual enzymes from the above system lead to the accumulation of the substrate and its distribution to all cell membranes interacting with lysosomes, and these are then called lysosomal storage disorders (LSD) (7). Due to the multidirectional effects of sphingolipids, disruption of their metabolism may be one of the risk factors for the development of diseases, including cardiovascular diseases (1). The aim of this review is to provide an overview of the current knowledge of sphingolipid metabolism and signaling in cardiovascular diseases such as hypertension, coronary artery disease, heart failure, arrhythmias, and stroke.

## Sphingolipid metabolism

Ceramide, a representative of biologically active sphingolipids, plays a fundamental role in the sphingolipid metabolic pathway (5). *De novo* synthesis is one of the three metabolic pathways leading to Cer synthesis (8). This process begins in the endoplasmic reticulum by the action of serine palmitoyltransferase (SPT) in the presence of the substrates: serine and palmitoyl-CoA or also possibly alanine or glycine and stearate or myristate, to produce 3-ketodihydrosphingosine (8). Subsequent reactions of the reduction by 3-ketodihydrosphingosine reductase, and N-acylation by a (dihydro)-ceramide synthase, and desaturation, generate Cer (9).

Apart from *de novo* biosynthesis, Cer can be generated in the cell through the hydrolytic pathway (5). Sphingomyelin is hydrolyzed by sphingomyelinase into phosphocholine and Cer (5).

And finally, ceramide may be produced from sphingosine *via* sphinganine N-acyltransferase (ceramide synthase) in the salvage pathway (1). In reverse, Cer may be hydrolyzed by ceramidase enzymes resulting in the product, sphingosine, which can be either phosphorylated by sphingosine kinases (SPHK1 and SPHK2) generating S1P or utilized in the salvage pathway (10). In addition, S1P lyase (SPL) is the enzyme involved in irreversible degradation of S1P into ethanolamine phosphate and hexadecenal (11).

Cer, generated in the endoplasmic reticulum, is then transported to the Golgi apparatus. Upon Cer phosphorylation by Cer kinase, C1P is formed, which in turn can be decomposed by C1P phosphatase or glycosylated by glucosylCer or galactosylCer synthases (10, 12). O-acylation of Cer provides a structure similar to a triacylglycerol (TAG), is stored in lipid droplets (13). In the Golgi apparatus, Cer is also metabolized to sphingolipids of higher complexity, such as glycosphingolipids (GSL) and sphingomyelins (SM) (14). GSL and SM are there after shuttled to the plasma membrane, where they are used as structural components (14). The degradation of GSL, catalyzed by subsequent reactions of specific hydrolases, yields glucosylceramide and galactosylceramide, which are in turn hydrolyzed by distinct β-glucosidases and β-galactosidases to produce Cer (15).

**FIGURE 1:**
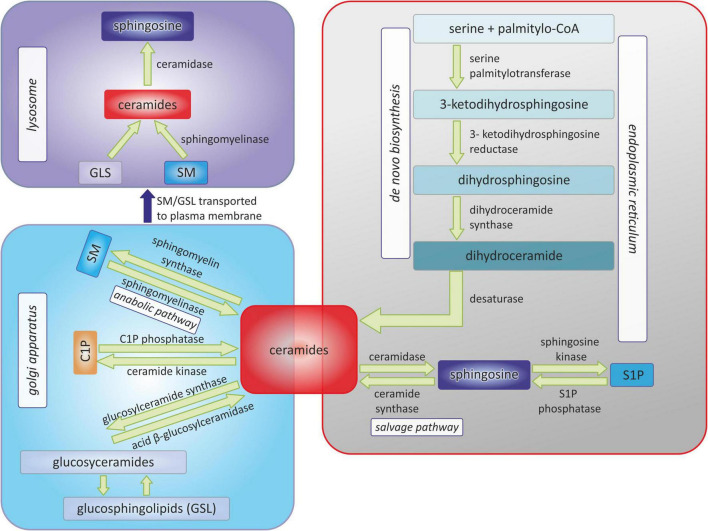
Pathways of sphingolipids metabolism. C1P, ceramide-1-phosphate; SM, sphingomyelin; S1P, sphingosine-1-phosphate.

Figure 1 shows schematically the metabolism of sphingolipids, due to the complexity of the processes, only the main molecules and enzymes are shown.

## Sphingolipids in the regulation of the cellular function

Sphingolipids play a major role in the regulation of the cellular function and influence almost all of the primary aspects of cell biology, including growth, the cell cycle, cell death, cell senescence, apoptosis, inflammation, the immune response, cell adhesion and migration, angiogenesis, nutrient uptake, metabolism, the response to stress stimuli, and autophagy (8).

Due to the fact that the article deals with the metabolism and signaling of sphingolipids in cardiovascular diseases, and most of the chapters are devoted to their involvement in heart diseases, this chapter will also discuss the role of sphingolipids in the regulation of cellular functions on the example of the heart muscle.

It has been demonstrated that the sphingolipid family is widely distributed throughout the heart muscle (1). In addition, sphingolipids perform many important physiological functions in the heart, among others, Cer and S1P were shown to directly regulate cardiomyocyte contractility. S1P has been proven to exert a negative inotropic effect on isolated mouse cardiomyocytes in the way of two intracellular pathways. First, works *via* G_*i*_ mediated which reduce L-type calcium channel current, blunt calcium-induced calcium release, and a decrease in the intracellular Ca_2_^+^ concentration. Second, acts *via* G_*i*_ decrease in activation of inwardly rectifying K^+^ current, consequently shortens the action potential duration (APD). A shorter APD leads to reduced calcium influx, decreased intracellular Ca^2+^ concentration, and therefore exerts negative inotropic effect (16). In turn, the addition of a cell-permeable Cer analog (C2-ceramide) to isolated rat cardiomyocytes has been shown to exert a positive inotropic effect due to enhanced sarcoplasmic reticulum calcium release and reabsorption as well as increased calcium sensitivity of the cardiomyocytes (17, 18). Moreover, treatment of isolated rat cardiomyocytes with C_6_-ceramide, which mimics endogenous ceramide generation and is considered to be more relevant to physiological conditions, decreased the peak amplitude of cell shortening without any influence on the peak Ca^2+^ transient. The observed negative inotropic response was mediated by the activation of the protein kinase C isoform ε, which is calcium-independent, and by the subsequent phosphorylation of cardiac troponin I (TnI) and cardiac myosin binding protein-C (cMyBP-C) (19). Based on the above data, it can be concluded that the influence of S1P and Cer on the force of heart contraction is opposite. While S1P weakens the force of muscle contraction (negative inotropic effect), Cer increases it (positive ionotropic effect). These data appear to be of particular interest for cardiovascular pharmacotherapy.

Sphingolipids are also involved in the pathways of myocardial fibrosis, which is one of the cornerstones of myocardial dysfunction in heart failure (HF). Pre-treatment of primary ventricular rat neonatal cardiac fibroblasts with dihydrosphingosine reduced transforming growth factor β (TGF-β)-induced collagen synthesis and increased the *de novo* synthesis pathway of dihydrosphingosine-1-phosphate, which on the other hand reduced the expression of the sphingosine-1-phosphate receptor 1 (S1PR1) (20). Administration of S1P to isolated cardiac fibroblasts resulted in increased α-smooth muscle actin (a marker of the transformation into collagen-producing myofibroblasts) and collagen expression in a sphingosine-1-phosphate receptor 2 (S1PR2)- and Rho kinase-dependent mechanism. Moreover, the authors have proven that TGF-β increases the expression and activity of SPHK1, whereas the blockage of SPHK1 and S1PR2 and the administration of anti-S1P monoclonal antibody completely blocks TGF-β-stimulated collagen production (21). Transgenic mice with overexpression of SPHK1 developed interstitial and perivascular myocardial fibrosis as well as sporadic calcification within the fibrotic areas, which was not observed in wild type mice. Transgenic mice also showed increased expression levels of collagen type I α1 and α2 proteins when compared with wild type animals. Sphingosine-1-phosphate receptor 3 (S1PR3) deletion partially inhibited cardiac fibrosis in transgenic mice with overexpression of SPHK1, therefore the authors concluded that SPHK1-dependent myocardial fibrosis involved the S1PR3-Rho family small G protein signaling pathway, and the release of reactive oxygen species and the activation of TGF-β (22). Moreover, S1PR1 transgenic mice showed hyperplastic fibroblasts and/or myofibroblasts, ventricular hypertrophy, and diffuse interstitial fibrosis without any hemodynamic stress. The above studies indicate that S1P promote myocardial fibrosis due to an activating effect on cardiac fibroblasts and myofibroblasts, which synthesize and release collagen into the extracellular matrix. This leads directly to interstitial fibrosis, cardiac hypertrophy and stiffness and my consequently cause HF.

Sphingolipids are also thought to mediate cardiomyocyte hypertrophy, although the results are ambiguous. Robert et al. reported that incubation of rat neonatal cardiomyocytes with S1P results in a concentration-dependent cardiomyocyte hypertrophy mediated by S1PR1 (23). On the other hand, Sekiguchi et al. revealed that S1P does not induce hypertrophy of neonatal rat cardiomyocytes, which was induced by sphingosylphosphorylcholine (SPC), however, SPC is not an agonist of S1P receptor, therefore the exact mechanism responsible for this observation is not yet fully understood (24).

## Sphingolipids in cardiovascular diseases

### Hypertension

According to the ESC/ESH Guidelines for the management of arterial hypertension from 2018, hypertension is defined as systolic blood pressure (SBP) ≥140 mmHg and/or diastolic blood pressure (DBP) ≥90 mmHg measured in a doctor’s office. Hypertension is a significant clinical problem. It is predicted that by 2025 the number of people with hypertension will increase to 1.5 billion (25).

The available data show the pivotal role of sphingolipids in the regulation of vascular tone and endothelial cells dysfunction (26–28). Therefore, it can be hypothesized that sphingolipids represent a new pathophysiological mechanism involved in the development of hypertension.

The above hypothesis seems to be confirmed by *in vitro* studies. In isolated bovine small coronary arteries and in human endothelial cells, Cer induced the production of reactive oxygen species (ROS) causing endothelial cell dysfunction and reducing bioactive nitric oxide (NO) (29, 30). In addition, in isolated carotid arteries from spontaneously hypertensive (SHR) rats shifting the Cer/S1P ratio toward ceramide dominance by the administration a sphingosine kinase inhibitor (dimethylsphingosine), and neutral sphingomyelinase contributed to endothelial-dependent vasoconstriction in which phospholipase A2, cyclooxygenase-1, and thromboxane synthase which was not observed in normotensive Wistar Kyoto rats (31). Additionally, it has been shown that Cer induced vasoconstriction in isolated canine cerebral arterial smooth muscle by increasing intracellular calcium concentration (32).

Moreover, *in vivo* studies seem to support the role of sphingolipids in the pathophysiology of hypertension. Namely, SHR rats were shown to have increased total ceramide (mainly C16:0, C22:0, C24:1, and C24:0) and sphingosine plasma concentrations (31). Based on the available data, it can be assumed that the plasma concentration of ceramides may be a potential marker of the early diagnosis of hypertension. It has been shown that endothelial deletion of SPTLC2 (long chain subunit 2 of serine palmitoyltransferase–the first enzyme of the *de novo* pathway) in mice revealed an important role of endothelial ceramides *de novo* biosynthesis in vascular and blood pressure homeostasis, and demonstrated that endothelium was a key source of plasma ceramides (33). In turn, Siedlinski et al. revealed that angiotensin II (AngII)-induced hypertension is associated with increased plasma concentrations of S1P in wild type (C57BL/6J) mice and chronic infusion of S1P caused a moderate, increase in systolic blood pressure, endothelial dysfunction, and vascular contractility in the mesenteric arteries (34). Meissner et al. showed that AngII increased the blood pressure in wild type mice, whereas in SPHK1 knockout (SPHK1^–/–^) mice the blood pressure response to AngII was significantly lower or even absent in the SPHK2 knockout (SPHK2^–/–^) mice (35). Similarly, Siedlinski et al. showed in SPHK1*^–/–^*mice that AngII infusion caused less advanced hypertension in comparison with wild type mice (34). Moreover, administration of SPHK2 antagonists, but not the SPHK1 antagonist, significantly reduced blood pressure in wild type mice injected with AngII (35). It was also shown that mice lacking Nogo-B (an endoplasmic reticulum membrane protein which inhibits serine palmitoyltransferase, present with hypotension) are resistant to AngII-induced hypertension and have preserved endothelial function and NO release (36).

While most of the data support a significant role of ceramide in the generation of hypertension, information regarding S1P in the pathogenesis of hypertension is inconclusive. Exogenous S1P appears to act as an activator of endothelial nitric oxide synthase (eNOS) through its receptors S1PR1 and S1PR3 (37, 38). S1P is also secreted from the endothelial cells and regulates flow-mediated vasodilation through the S1P-S1PR1 autocrine signaling axis (39). However, in greater concentrations, exogenous S1P was shown to induce vasoconstriction in resistance arteries mediated predominantly by S1PR3 and S1PR2 receptors (40, 41). Nevertheless, it has been suggested that activation of eNOS through S1PR1 and S1PR3 neutralizes S1PR2 and S1PR3-mediated vasoconstriction in vascular smooth muscle cells (26). In addition, Hu et al. (42) revealed that mice with S1PR1 knockout specifically in renal collecting ducts have reduced urinary sodium excretion, impaired pressure natriuresis, and developed more severe hypertension after 10-day deoxycorticosterone acetate (DOCA) salt treatment in comparison with wild type mice. Therefore, the authors concluded that collecting duct-S1PR1 signaling can play a significant role as an antihypertensive pathway by the induction of sodium excretion and they suggested that the dysfunction of the renal medullary S1PR1 could be a novel mechanism for salt-sensitive hypertension (42). It has also been shown that mice lacking endothelial S1PR1 had higher systolic blood pressure than the controls, had reduced S1P- and flow-mediated vasodilation, had reduced basal and acetylcholine (Ach)-induced nitric oxide production in the mesenteric arteries, and had lower plasma levels of nitrite. Therefore, the S1P-S1PR1-NO axis played an important role in the flow-mediated regulation of vascular tone and therefore the regulation of blood pressure (43).

Clinical studies seem to confirm the possibility of using sphingolipids as markers of arterial hypertension. Hypertensive patients also had significantly higher total plasma levels of Cer (mainly C24:1 and C24:0) and S1P than normotensive controls (31). Jujic et al. analyzed the family based cohort of Malmö Offspring Study and revealed that patients who had systolic blood pressure ≥140 mmHg had significantly higher plasma concentrations of S1P in comparison with patients with systolic blood pressure below 120 mmHg (44). Moreover, it has been shown that plasma concentrations of S1P were significantly correlated with impaired endothelial function assessed by the Reactive Hyperemia index and a higher aortic systolic pressure (34). In addition, Zheng et al. showed that plasma SM concentrations in patients with hypertension but without a physiological nocturnal reduction of ≥10% of blood pressure (non-dippers) was significantly higher in comparison with the dipper group and plasma SM levels were negatively correlated with the degree of systolic and diastolic blood pressure decrease at night (45).

Interestingly, both experimental and clinical studies have shown that hypertensive treatment may interact with sphingolipid metabolism. Administration of fingolimod (FTY720), which is a non-selective S1PR1, 3, 4, 5 agonist, increased the blood pressure in normotensive C57BL/6 mice and exacerbates hypertension in AngII mouse model, underlining the antihypertensive function of S1PR1 signaling (35, 43). Acute administration two differentially selective S1P agonists, FTY720 and BAF312 (more selective S1P1,5 agonist) to Sprague Dawley rats resulted in S1P1-mediated bradycardia. However, administration of FTY720, but not BAF312, resulted in caused dose-dependent hypertension mediated by S1PR3 (46). In addition, oral administration of FTY720 in SHR rats, but not in Wistar Kyoto rats, increased the mean arterial pressure and induced large contractions in isolated carotid arteries, most probably due to the inhibition of sphingosine kinase (47). Spijkers et al. (48) have shown that treatment of SHR rats with losartan (Ang II type 1 receptor antagonist) or hydralazine (the non-selective vasodilator) not only reduced blood pressure, but also decreased vascular Cer concentration and improved endothelial-dependent vasorelaxation of isolated carotid arteries. Moreover, only losartan reduced sphingomyelinase-induced contractions of isolated carotid arteries, which may result from the reduction in the endothelial expression of calcium-independent phospholipase A2 (48). Furthermore, S1P has been associated with a higher baseline plasma renin activity and a reduced blood pressure response to hydrochlorothiazide (thiazide diuretic) in hypertensive patients (49).

### Coronary artery disease

#### Atherosclerosis and chronic coronary syndromes

Atherosclerosis is a pathophysiological mechanism underlying coronary artery disease (CAD). Atherosclerotic plaques are formed within the regions of disrupted endothelium and/or the arterial wall. Within the bloodstream, lipids attach to water-soluble lipoproteins named apolipoproteins. Lipoproteins containing apo-B, which are the major protein component of very low-density lipoprotein (VLDL), intermediate density lipoprotein (IDL), and low-density lipoprotein (LDL), pass through the disrupted endothelium and are endocytosed by macrophages within the intimal layer of the arteries. Therefore, lipoproteins are oxidized, which induces leukocyte accumulation and the formation of foam cells. It has been shown that LDL aggregation promotes the accumulation of cholesteryl ester-rich lipid droplets in the human primary monocyte-derived macrophages, induces matrix metalloproteinase-7 secretion from macrophage foam cells, and activates T-cells *in vitro* (50). LDL aggregation susceptibility depends on the particle lipid composition, including higher concentrations of sphingomyelins and ceramides and may be decreased by diet or proprotein convertase subtilisin/kexin 9 (PCSK9) inhibition (50). Moreover, blood flow disturbance within atherosclerosis-susceptible regions promotes the rearrangement of the microenvironment of the arterial wall intima, with vascular smooth muscle cell migration, extracellular matrix remodeling, plaque growth, fibrous cap formation, and necrotic core formation with calcifications (51, 52). Interestingly, LDL aggregation susceptibility prognosticates cardiovascular death in patients with CAD, therefore it has been concluded that intimal LDL aggregation facilitates atherogenesis, inflammation, and plaque rupture by inducing foam cell formation and the secretion of matrix metalloproteinase-7, and by activating T-cells, which increases the risk of death (50). Therefore, the reduction of sphingolipid content in aggregation-prone LDL with diet or treatment with a PCSK9 inhibitor could be beneficial in reducing the risk of CAD (50). Another important predisposing factor for the development of atherosclerosis is the deposition of the mineral hydroxyapatite in the extracellular matrix and in the arterial smooth muscle cells, leading to arterial calcification, which in turn is associated with plaque rupture, and an increased risk of heart disease and stroke (53). The calcification process can affect both the inner (arterial intimal calcification; AIC) and the middle (arterial medial calcification; AMC) walls of the arteries. AIC is associated with the obstruction of the arteries and the rupture of the atherosclerotic plaque (54), whereas, AMC predisposes to vessel stiffness and the development of systolic hypertension and increased pulse wave velocity, consequently leading to diastolic dysfunction and heart failure (55). Yuan et al. (56) showed that endothelium-specific AC gene knockout mic (Asah1*^fl/fl^*/EC*^cre^*) with streptozotocin-induced diabetes significantly augmented the production and activation of NOD-like receptor pyrin domain 3 (NLRP3) inflammasomes in the coronary artery endothelial cells, as well as significantly higher expression of exosome markers auch as CD63 and alkaline phosphatase (ALP) in the coronary arterial wall and interleukin 1 beta (IL-1β) release of exosomes in comparison with wild-type (WT/WT) littermates. Moreover, Asah1*^fl/fl^*/EC*^cre^* mice trated with streptozotocin revealed significant thickening of the coronary arterial wall and lower expression of tight junction protein compared to WT/WT littermates (56). Additionally, it has been shown that smooth muscle-specific acid ceramidase (Ac) gene knockout mice (Asah1*^fl/fl^*/SM*^Cre^*) during hypercalcemia caused by high doses of vitamin D had more severe AMC in both the aorta and the coronary arteries as well as upregulation of osteopontin and RUNX2 (osteogenic markers) in the aortic and coronary arterial medial wall, and CD63, AnX2 (sEV markers) and ALP expression (mineralization marker) in the coronary arterial media in comparison with Asah1*^fl/fl^*/SM*^wt^* and WT/WT mice (57). In addition, the same researchers showed that high doses of vitamin D-induced hypercalcemia in Smpd1*^trg^*/SM*^cre^* mice with overexpressing the acid lysosomal sphingomyelinase (SMPD1) gene specific for aortic and coronary smooth muscle cells contributed to higher aortic and coronary AMC as well as increased expression of RUNX2 and osteopontin in the coronary and aortic media (58).

On the basis of the available studies, it can be hypothesized that sphingolipids play an important role in endothelial dysfunction and thus may promote the atherosclerotic processes.

**FIGURE 2:**
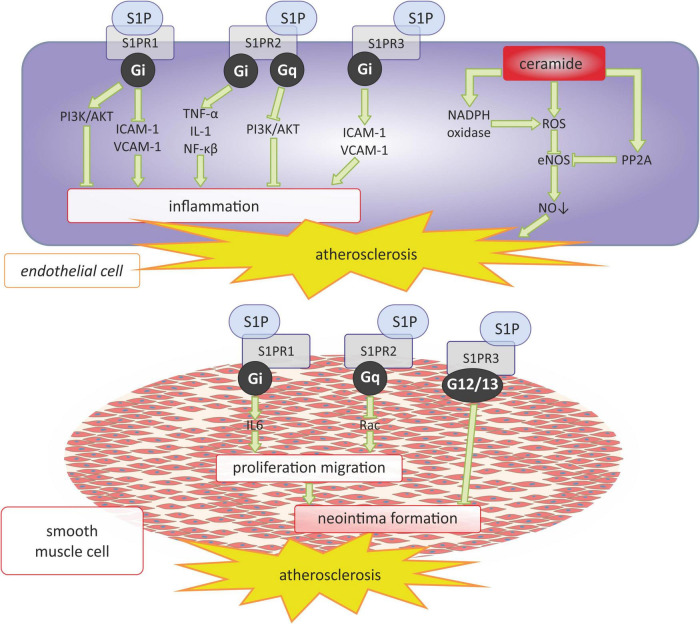
Sphingosine 1-phosphate and ceramide signaling in atherosclerosis. AKT, serine/threonine protein kinase B; eNOS, endothelial nitric oxide synthase; G_*i*_, G_*i*_ protein alpha subunit; G_*q*_, G_*q*_ protein alpha subunit; ICAM-1 -intercellular adhesion molecule 1; IL-1, interleukin 1; IL-6, interleukin 6; NADPH oxidase, nicotinamide adenine dinucleotide phosphate oxidase; NF-κB, nuclear factor kappa-light-chain-enhancer of activated B cells; NO, nitric oxide; PI3K, phosphoinositide 3-kinase; PP2, protein phosphatase 2, Rac, family small GTPase; ROS, reactive oxygen species; S1P, sphingosine-1-phosphate; S1PR1, sphingosine-1-phosphate receptor 1; S1PR2, sphingosine-1-phosphate receptor 2; S1PR3, sphingosine-1-phosphate receptor 3; TNF-α, tumor necrosis factor α; VCAM-1, vascular cell adhesion molecule 1.

S1P appears to have a twofold effect on the development of atherosclerosis depending on the receptors through which it acts (59) (Figure 2). The interaction of S1P with S1PR1 may have antiatherosclerotic effects by inhibiting macrophage apoptosis and endothelial inflammation *via* intracellular pathway dependent on the phosphatidylinositol 3-kinase (PI3K) and protein kinase B (PI3K/Akt) (60). On the other hand, S1PR1 may have a positive effect on the development of atherosclerosis by activating pro-inflammatory endothelial factors such as: intercellular adhesion molecule (ICAM-1) and vascular cell adhesion molecule 1 (VCAM-1), as well as by facilitating the formation of neointima formation by increasing the level of interleukin 6 (IL-6), which stimulates the proliferation and migration of vascular smooth muscle cells (VSMCs) (61, 62). S1P interacting with S1PR2 may also have a pro-atherosclerotic effect by promoting the inflammatory response of endothelial cells due to the increased expression of pro-inflammatory factors such as tumor necrosis factor α (TNF-α), interleukin 1 (IL-1), stimulating endothelial dysfunction (63). However, the interaction of S1P with S1PR2 may also have antiatherosclerotic effects by inhibiting the proliferation and migration of smooth muscle cells *via* the Rac protein and delaying the neointima formation (64, 65). Skoura et al. (66) have shown that ApoE and S1PR2-deficient *Apoe-/-S1pr2-/-*mice show reduced development of western diet-induced atherosclerosis, present decreased fibrous tissue, collagen and lipid content, as well as decreased necrotic core within the atherosclerotic plaque and a reduced number of macrophages within the vessel wall in comparison with the *Apoe-/-S1pr2* + / + controls. Therefore, the authors suggested that S1PR2 signaling regulates macrophage infiltration and inflammatory cytokine secretion, thereby promoting atherosclerosis (66). On the other hand, Guanbataar et al. showed that ApoE-deficient mice receiving a S1PR2 antagonist and fed on a western-type diet resulted in the reduction of atherosclerotic plaque development, and alleviated endothelial dysfunction as well as resulting in significantly decreased lipid deposition, macrophage accumulation, and the expression of inflammatory proteins within the aorta (67). Similarly, S1P by activating S1PR3 can also promote the development of atherosclerosis, as does S1PR1 by activating endothelial ICAM-1 and VCAM-1 and causing its dysfunction (68). But S1P/S1PR3 signaling may also have antiatherosclerotic effects by inhibiting neointima formation (69). This same authors shown that S1PR3 genetic deficiency significantly increased neointima formation in the mouse carotid artery ligation model (69).

Interestingly, the available data indicate that not only the interaction of S1P with its receptors, but also the enzymes leading to S1P synthesis, may play an important role in the pathogenesis of atherosclerosis. Ishimaru et al. revealed that SPHK2, but not SPHK1-deficient mice, developed larger atherosclerotic plaques in comparison with the control mice, with no influence on the plasma lipid profile (70). Moreover, the authors found that SPHK2 is responsible for autophagosome- and lysosome-mediated catabolism of the intracellular lipids, therefore SPHK2 has been suggested to be a novel target for the treatment of atherosclerosis (70). The above observations seem to be corroborated by Feuerborn et al. who showed that LDL receptor-deficient mice transplanted with bone marrow lacking SPKH2 have an increased concentration of S1P in the erythrocytes, plasma, and high-density lipoprotein (HDL), and a reduced development of atherosclerotic plaques with smaller necrotic cores within lesions (71). Potì et al. revealed that, in LDL receptor-deficient mice on a western high-cholesterol diet, lowering plasma S1P levels by treatment with SKI-II, a SPHK1 inhibitor, increased the development of atherosclerotic lesions, and increased the levels of TNF-α and the expression of endothelial adhesion molecules (soluble intercellular adhesion molecule-1, sICAM-1; soluble vascular cell adhesion molecule-1, sVCAM-1) (72).

Similar to the enzymes that influence S1P synthesis, sphingomyelinases, which play a role in the anabolic pathways leading to the formation of ceramides from sphingomyelin, appear to play an important role in the pathogenesis of atherosclerosis. For example, it was shown that neutral as well as acid sphingomyelinase, which are expressed in the intima of atherosclerotic arteries, promotes foam cell formation by the production of Cer in lipoproteins and the enhancement of LDL uptake and has the ability to induce cholesteryl ester accumulation in macrophages (73–75). Moreover, sphingomyelinase induces aggregation and fusion of LDL, which can be a potential mechanism responsible for the focal retention of extracellular lipids in the arterial wall (76, 77). Moreover, ceramides themselves exert a pro-atherosclerotic effect by inducing endothelial dysfunction. It has been shown that ceramides in endothelial cells generate the ROS production and activating protein phosphatase 2A (PP2A), and consequently reduce the activity of eNOS, thus impairing the production and bioavailability of NO (28) (Figure 2).

The activity of sphingomyelin synthases also plays an important role in the pathogenesis of atherosclerosis. In ApoE-deficient mice, the administration of an adenovirus containing sphingomyelin synthase 2 resulted in the increased development of atherosclerosis, and its overexpression is associated with an increased aortic inflammatory response, contributing to endothelial dysfunction and plaque instability (78, 79). In addition, the overexpression of sphingomyelin synthases 1 and 2 significantly decreased (high-density lipoprotein) HDL-sphingomyelin and HDL-cholesterol, and increased non-HDL-sphingomyelin, non-HDL-cholesterol, and apolipoprotein B concentrations, thus exerting a proatherogenic effect (80).

**FIGURE 3:**
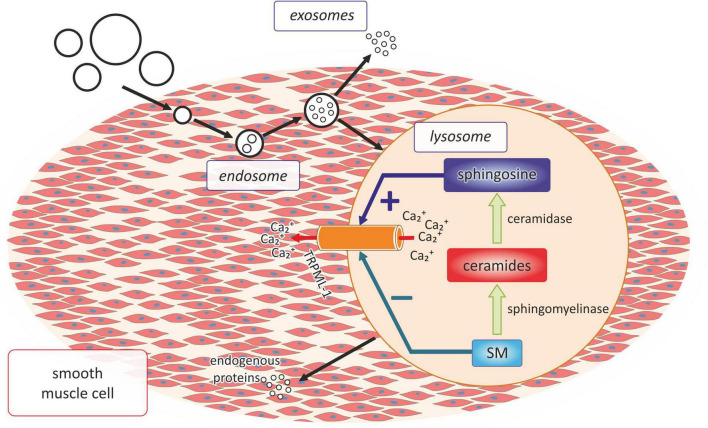
The role of lysosomal sphingolipid in the process of vascular calcification. SM, sphingomyelin; Ca_2_^+^, calcium ions.

Moreover, the latest research also indicates the important role of sphingolipids in the process of arterial calcification, which is one of the main mechanisms leading to the development of atherosclerosis (53) (Figure 3). The research of Koka et al. conducted both in the primary cultures of the mouse carotid arterial endothelial cells as well as in the mouse model of hypercholesterolemia showed that acid sphingomyelinase and ceramidases related to the formation of the membrane raft were necessary for the activation of NRLP3 inflammasomes in the endothelial cells and further increased atherosclerosis associated with hypercholesterolemia (81).

Numerous experimental data indicate the beneficial effect of modulating sphingolipid system in the pharmacotherapy of atherosclerosis. Exogenous administration of fingolimod decreased the development of atherosclerosis in LDL receptor-deficient mice and ApoE-deficient mice (82, 83). Administration of KRP-203, a S1PR1 agonist, reduced the development of atherosclerosis in LDL receptor-deficient mice on a cholesterol-rich diet by modulating the lymphocyte and macrophage function and improving the endothelial barrier (84). In ApoE-deficient mice on a chow or high fat diet, inhibition of *de novo* ceramide synthesis by the administration of myriocin, an inhibitor of SPT, resulted in significantly decreased plasma concentration of sphingomyelin, Cer, and S1P, and a significant increase in plasma phosphatidylcholine levels with no significant change in the cholesterol and triglyceride plasma concentrations. Most interestingly, both in ApoE-deficient mice fed on a chow or high fat diet, the administration of myriocin resulted in a significantly reduced atherosclerotic lesion area (85). In other studies on ApoE-knockout mice fed on a western diet, treatment with myriocin decreased plasma concentrations of sphingomyelin, sphinganine, cholesterol, and triglyceride with a concomitant decrease in beta-very-low-density lipoprotein (beta-VLDL) and LDL cholesterol, an increase in HDL cholesterol, a significant reduction of arterial atherosclerosis development, a reduction in the lesion and macrophage area, and the inhibition of necrotic core formation (86, 87).

Clinical studies seem to confirm the important role of sphingolipids in the atherogenic process and in the pathogenesis of CAD. However, data on the concentration of non-HDL-bound S1P in patients with CAD were inconclusive, but they were consistent with the decreased concentration of HDL-bound S1P in this group of patients, suggesting that CAD is associated with an uptake in the disturbance of HDL for plasma S1P (88–90). Moreover, HDL-associated S1P concentrations showed negative correlation with the severity of CAD and distinguishes one-vessel disease from multi-vessel disease, and a low concentration of HDL-associated S1P was predictive for CAD advancement (89). The serum concentration of S1P has been shown to be more predictive of obstructive CAD in comparison with traditional risk factors, such as age, sex, a family history of CAD, diabetes mellitus, lipid profile, and hypertension (90). Nevertheless, patients with stable CAD have increased plasma concentrations of sphingolipids, for example: ceramides, sphingomyelins, sphinganine, and sphingosine (91, 92). In a study by Poss et al., the total C24:1-containing sphingolipids and/or total dihydroceramides, independent of the chain length, were most strongly associated with the occurrence of CAD (91). Higher levels of plasma Cer(d18:1/20:0), Cer(d18:1/22:0), and Cer(d18:1/24:0) were significantly associated with the presence of left anterior descending coronary artery (LAD) stenosis ≥50% (93). Also, a high ratio of Cer(d18:1/24:1) to Cer(d18:1/24:0) was shown to be associated with more severe coronary artery stenosis (94). Finally, Meeusen et al. revealed that increased plasma concentrations of Cer(16:0), Cer(18:0), and Cer(24:1) are independently associated with major adverse cardiovascular events in patients with and without CAD (95). Moreover, a recent meta-analysis has shown that higher plasma concentrations of Cer(d18:1/16:0), Cer(d18:1/18:0), and Cer(d18:1/24:1) were associated with major adverse cardiovascular events (96). Therefore, novel CAD predictive ceramide risk scores were proposed, which show superior performance in comparison with traditional cardiovascular risk markers (91, 93, 97–100). Hilvo et al. improved the previously proposed ceramide risk score (CERT1) and established CERT2, which is a ceramide-based and phospholipid-based risk score for predicting the residual CVD event risk, especially cardiovascular death, in patients with CAD (97). CERT2 has been shown to have superior prognostic efficacy over troponin T (TnT) and LDL cholesterol and is associated with biomarkers of inflammation, myocardial necrosis, myocardial dysfunction, renal dysfunction, and dyslipidemia (97, 101).

In addition, based on available clinical studies, it appears that Cer composition seems to influence the morphology and vulnerability of atherosclerotic plaque. In the ATHEROREMO-IVUS study, Cheng et al. (102) revealed that higher plasma concentrations of Cer(d18:1/16:0) and Cer(d18:1/24:0) were associated with higher necrotic core fractions and lactosylceramide (d18:1/18:0), and with a higher coronary lipid core burden index (LCBI) on near-infrared spectroscopy (NIRS). A higher Cer(d18:1/20:0)/Cer(d18:1/24:0) ratio was associated with a higher plaque burden and the presence of dense calcium (102). Pan et al. (103) performed Optical Coherence Tomography in patients with STEMI and correlated culprit lesion characteristics with plasma Cer concentrations. The authors revealed that lipid-rich plaque, plaque rupture, and thin-cap fibroatheroma were more prevalent, while calcification occurred less often in patients with a high plasma Cer concentration in comparison with individuals with lower plasma Cer levels (103). Patients with a high plasma Cer concentration had a longer lipid core length, a larger mean lipid arc, and a bigger lipid volume index, which positively correlated with plasma Cer(d18:1/16:0), Cer(d18:1/18:0), and Cer(d18:1/24:1), and a thinner fibrous cap thickness (negatively correlated with the abovementioned Cer species) when compared with the low plasma Cer group. Moreover, four Cer species which have previously been shown to have a strong predictive value for major adverse cardiovascular events [Cer(d18:1/16:0), Cer(d18:1/18:0), Cer(d18:1/24:1), and Cer(d18:1/24:0)] were also predictors for thin-cap fibroatheroma (103).

#### Myocardial ischemia/reperfusion injury

The current treatment of choice for acute myocardial ischemia is based on the immediate restoration of blood flow using primary coronary intervention (PCI) or thrombolytic therapy. Paradoxically, such early and fast restoration of blood flow further increases the ischemia-induced myocardial injury, which is called myocardial ischemia/reperfusion injury (IRI) (104–106).

Numerous studies have revealed that ischemic hearts show an increase in Cer and S1P levels, therefore it can be concluded that sphingolipids are involved in the pathophysiology of IRI. However, considering the important role of ceramides in apoptosis of cardiomyocytes, researchers most often attribute an unfavorable effect on the myocardium to them, while S1P is considered a cardioprotective effect.

As mentioned above, Cer mediates the ischemia/reperfusion-induced apoptosis of cardiomyocytes (107, 108). *In vitro* studies on isolated rat hearts subjected to ischemia/reperfusion (I/R) have an increased myocardial level of Cer and S1P, and decreased levels of sphingomyelin and sphingosine (109–111). Moreover, it was found that in isolated rat hearts subjected to I/R, Cer was accumulated specifically in the mitochondria, but not in the microsomal fraction (112). The I/R group also had increased mitochondrial activity of neutral sphingomyelinase, which was decreased in rats treated with a sphingomyelinase inhibitor (D609), with no changes in the microsomal fraction in any group. Moreover, in isolated rat hearts subjected to I/R, the injection of D609 significantly reduced infarct size, myocardial ceramide concentration, and the ratio of active caspase 3/pro-caspase 3, as a marker of active apoptosis and ROS production (112). Additionally, ischemic pre-conditioning seems to partially prevent a reperfusion-induced increase in myocardial Cer concentrations (113).

Moreover, *in vivo* studies have shown that increased myocardial concentrations of ceramide due to I/R are probably associated with the reduced activity of ceramidase (111). A rat model of I/R revealed that the ischemic myocardium as well as the ischemic and reperfused myocardium have an increased concentration of Cer(16:0, 18:0, 18:1, 18:2, and 20:4) (108, 113). Additionally, it was revealed that I/R in mice increased the myocardial concentration of ceramide both in the infarct area and in the area at risk, with a concomitant increased expression of two subunits of the SPTLC1 and SPTLC2 in the area at risk, suggesting that I/R enhances the accumulation of ceramide *via* the activation of the *de novo* synthesis pathway (114). In turn, intraventricular administration of nanocarriers loaded with the SPT inhibitor myriocin at reperfusion significantly reduced the infarct size, decreased the inflammatory response in the area at risk, and reduced the myocardial reactive oxygen species production (114). Since myriocin also regulates cardiac metabolism and inflammatory responses after I/R injury, it has been proposed that myriocin might serve as a post-conditioning therapeutic tool (115).

Whereas Cer has a rather an adverse effect on the heart muscle, S1P protected the heart from I/R myocardial injury and is released together with adenosine during ischemic pre-conditioning and post-conditioning (116–118). Pre-conditioning or post-conditioning with S1P protected the isolated rat heart against I/R-induced myocardial injury (119–121). This is probably done *via* controlling the mitochondrial function and Akt/glycogen synthase kinase β (Gsk3β) phosphorylation (121). Moreover, S1P played a cardioprotective role in I/R also through the activation of the S1PR3- Ras homolog family member A/protein kinase D (RhoA/PKD) signaling cascade in S1P3 KO mouse cardiomyocytes (122). Moreover, pharmacological inhibition of S1P lyase prior to I/R in isolated murine hearts increased the myocardial levels of S1P, reduced the infarct size and enhanced hemodynamic recovery Moreover, pharmacological inhibition of S1P lyase prior to I/R in isolated murine hearts increased the myocardial levels of S1P, reduced the infarct size and enhanced hemodynamic recovery (123). On the other hand, studies on isolated mouse and rat hearts showed that ischemia decreased the myocardial activity of sphingosine kinase and activates S1P lyase, which leads to reduced myocardial levels of S1P (123, 124).

S1P can affect the myocardium during I/R through its receptors. Studies carried out on both mouse and rat hearts have shown that exogenously administered S1P and stimulation of the S1PR1 receptor, as well as activation of SPHK1, protect against hypoxia- and oxygen-glucose deprivation/reoxygenation-induced myocardial cell death, respectively (125–128). Furthermore, in mouse model of I/R, S1P reduced the myocardial infarction size, reduced inflammatory cell recruitment and apoptosis of cardiomyocytes, thus exerting cardioprotective effects against I/R injury, through the activation of S1PR3-mediated and nitric-oxide dependent pathways (129). Additionally, Means et al. showed that activation of both S1PR2 and S1PR3 receptors are involved in cardioprotection against I/R injury, since the knockout of either S1PR2 or S1PR3 alone does not influence the *in vivo* response to I/R injury, while the knockout of both receptors significantly increases the I/R injury-related infarct size (130). Recently, Zhang et al. (131) in the rat model of I/R showed that blockage of the S1PR2/S1PR3 receptors with specific antagonists increased IR-induced mortality and infarction size, induced conduction abnormalities, and decreased the fractional shortening and ejection fraction of LV. While pre-treatment with S1PR2 and S1PR3 agonists at least partially reversed those changes (131). Afterward, the interaction of S1P with its receptors activates intracellular pathways also having a cardioprotective effect against ischemia. It have been shown that two signaling pathways to be involved in the above process: the Survivor Activating Factor Enhancement (SAFE) and the Reperfusion Injury Salvage Kinase (RISK) pathways. The SAFE pathway is activated by ischemic conditioning and/or by reperfusion and promotes cell survival through the activation of TNF-α which therefore activates its receptor TNF-R2, leading to the Janus kinase/signal transducer and activator of transcription 3 pathway (JAK/STAT-3) (132). The RISK pathway is activated by reperfusion and has a cardioprotective effect through the activation of two independent cascades, i.e., the PI3K-Akt or mitogen-activated extracellular signal-regulated kinase (MAPK, MEK1/ERK1/ERK2) (132). Somers et al. (133) revealed that S1P administered at the time of reperfusion to isolated rat hearts subjected to I/R reduced the myocardial infarct size in wild type animals, but not in STAT-3 or TNF-deficient mice. Moreover, after reperfusion in wild type mice, the authors revealed increased nuclear levels of phosphorylated STAT-3, Akt, and FOXO1, which is a downstream target of the RISK pathway (133). Moreover, it has been shown that activation of STAT-3 is required for the S1P-induced reduction of the infarction area in I/R (134).

Although human studies regarding sphingolipid involvement in I/R are scarce, it has been shown that, in patients undergoing elective percutaneous coronary intervention, coronary sinus levels of S1P, sphingosine, and sphinganine were increased as soon as 1 min following transient ischemia and reperfusion (135).

#### Acute coronary syndromes

Regarding the currently used nomenclature, acute coronary syndromes (ACS) include ST-segment elevation ACS. Most patients will develop ST-segment elevation myocardial infarction (STEMI) or non-ST-segment elevation ACS (NSTE-ACS), including myocardial ischemia without cardiomyocyte necrosis (unstable angina) or non-ST-segment elevation myocardial infarction (NSTEMI) (106). From the pathophysiological point of view, myocardial infarction (MI) mostly results from atherosclerotic plaque rupture or erosion and in the vast majority of cases depends on the composition and vulnerability of the atherosclerotic plaque (51).

Based on the available data, it can be concluded that sphingolipids also play an important role in the pathogenesis of acute coronary syndrome and myocardial infarction. Moreover, the studies presented below reveal a rather unfavorable effect of Cer on the heart muscle and a definitely cardioprotective effect of S1P.

**FIGURE 4:**
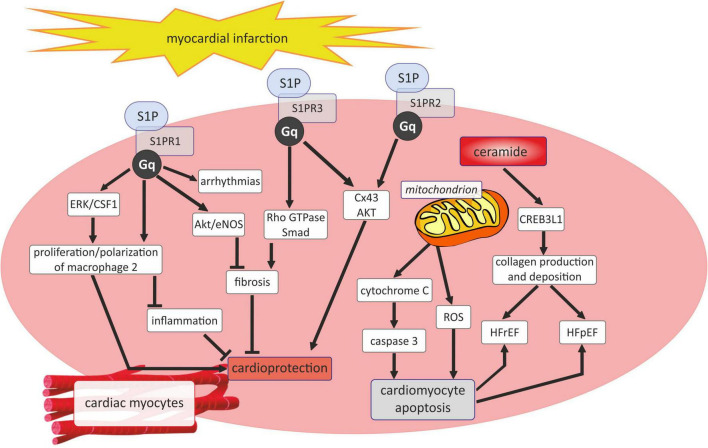
Sphingosine 1-phosphate and ceramide signaling in myocardial infarction. AKT, serine/threonine protein kinase B; CREB3L1, cyclic adenosine monophosphate responsive element binding protein 3 like 1; CSF1, colony stimulating factor 1; Cx43, connexin43; ERK, extracellular signal-regulated kinases; G_*q*_, G_*q*_ protein alpha subunit; HFpEF, heart failure with preserved ejection fraction; HFrEF, heart failure or heart failure with reduced ejection fraction; Rho GTPase, family small GTPase; ROS, reactive oxygen species; SMAD -suppressor of mothers against decapentaplegic; S1P, sphingosine-1-phosphate; S1PR1, sphingosine-1-phosphate receptor 1; S1PR2, sphingosine-1-phosphate receptor 2; S1PR3, sphingosine-1-phosphate receptor 3.

It has been shown that ceramides by their activity in mitochondrial membranes caused alter cellular energetics, generating ROS formation and initiating cytochrome C release to promote apoptosis through caspase 3 pathways (Figure 4) (28). Cer also play a profibrotic role, because they can active of cyclic adenosine monophosphate responsive element binding protein 3 like 1 (CREB3L1) pathway to promote collagen deposition consequently leading to the development of heart failure with reduced ejection fraction (HFrEF) as well as heart failure with preserved ejection fraction (HFpEF) (Figure 4) (28).

The adverse effects of Cer on the heart were reflected in *in vivo* experimental studies. Hadas et al. (136) have proven that, in Swiss Webster (CFW) mice 24 h after ligation of LAD, several genes involved in *de novo* synthesis of Cer were upregulated and myocardial concentrations of C16, C20, C20:1, and C24 ceramides were increased. Moreover, treatment with modified mRNA (modRNA) enhanced the activity of acid ceramidase and reduced hypoxia-induced cell death and resulted in a reduced inflammatory response, in improved cardiac function, in a smaller scar area, and in longer survival post infarction (136). In the reparative phase post MI induced by LAD ligation, C57BL/6 mice have significantly higher myocardial concentrations of Cer16:0, Cer24:1, ceramide-1-phosphates (Cer1P; 14:0, 18:1, 18:2, 18:3), dihydroceramide-1-phosphates (14:0, 16:0, 18:2, 20:4, 20:5), monohexosylceramides (20:0, 22:0, 24:0, and 24:1), dihexosylceramides (22:0, 24:1, 24:0), and lower myocardial concentrations of two dihydro sphingomyelins (38:4 and 40:0) in comparison with the sham-operated control group (137). Moreover, the MI group had higher expressions of Cer kinase (Cerk), as well as higher ratios of the Cer1P/Cer, which suggests that in the reparative phase post MI, the upregulated expression of Cerk may lead to enhanced conversion of Cer to Cer1P (137). In addition, myocardial concentrations of Cer(d18:1/16:0), Cer(d18:1/18:0), Cer(d18:1/20:0), Cer(d18:1/22:0), and Cer(d18:1/24:0), as well as the expression of serine palmitoyl transferase-2 (the gene encoding a base subunit of SPT), ceramide synthase 6 (CerS6), and neutral sphingomyelinase (nSMase) were found to be significantly higher in Wistar rats with acute MI induced by LAD occlusion in comparison with the sham-operated control group (138).

As mentioned before, unlike Cer, S1P is assigned a rather cardioprotective role in the course of acut coronary syndrome and myocardial infarction. It has been shown that the beneficial effects of S1P on the heart can be exerted by its receptors, of which S1PR1 and S1PR3 are widespread, while S1PR2 expression is low in the myocardium (Figure 4). S1PR1 has been disclosed to act as a protector in ischemic myocardial injury since soothes inflammatory responses and stimulates the proliferation of repair macrophages as well as inhibits myocardial fibrosis *via* Akt/eNOS dependent pathway (59). Nevertheless, S1PR1 also affect negatively on the heart by aggravating arrhythmias (59). In turn, the interaction of S1P with S1PR2 and S1PR3 exerts a cardioprotective effect, mainly by maintaining the conduction of myocyte action potential by inducing connexin 43 (Cx43) phosphorylation and phosphorylation of serine/threonine protein kinase B (AKT) (59). Similarly, the available experimental studies show a beneficial effect of S1P on the heart muscle in the course of MI. In C57BL/6 mice, pharmacological elevation of circulating S1P attracted hematopoietic stem cells and promoted tissue regeneration within the infarct zone, thus leading to cardiac structural and functional improvement after myocardial infarction (139). The studies of Yang et al. revealed that S1P induced the autophagy of cardiomyocytes after MI through the inhibition of the mammalian target of rapamycin (mTOR) signaling pathway, preventing adverse remodeling following MI in Sprague-Dawley rats (140). Moreover, in rats, myocardial infarction reduced the plasma concentrations of S1P, dihydrosphingosine-1-phosphate, sphingosine, and dihydrosphingosine but increased the concentration of total Cer, with concomitant alteration in the sphingolipid levels in the erythrocytes and platelets (141).

Based on the available data, it can be concluded that clinical trials seem to confirm the experimental results. Recently Yao et al. has shown that the Cer(d18:1/24:1(15Z)/Cer(d18:1/24:0) ratio, Cer(d18:1/14:0), and Cer(d18:1/22:0) can be independent predictors of acute coronary syndrome in patients with chest pain and added to high-sensitive troponin and traditional factors to improve the diagnostics of ACS (142). Patients with acute coronary syndrome, including unstable angina (UA) and AMI, had significantly higher concentrations of ceramide and secretory acid sphingomyelinase in comparison with healthy controls and individuals with stable angina (103, 143). Interestingly, increased plasma concentrations of Cer was sustained until a day after percutaneous coronary intervention in UA or day 7 in AMI patients (143). In another study, patients with ACS have been shown to have higher plasma concentrations of Cer(d18:1/16:0), Cer(d18:1/24:0), lactosylceramide (d18:1/18:0), and the Cer(d18:1/16:0)/Cer(d18:1/24:0) ratio in comparison with stable CAD (102). Recently, Burrello et al. revealed that patients with STEMI have significantly increased extracellular vesicle total content of Cer, dihydroceramides, and sphingomyelins in comparison with the controls and the prognostic ability of their levels was not inferior to troponin as a biomarker for AMI (144). The sphingolipid concentration within the extracellular vesicles (EV) was correlated to the peak level of high sensitive troponin and decreased 24 h post coronary artery reperfusion (144). Karagiannidis et al. evaluated patients with STEMI subjected to primary percutaneous coronary intervention and thrombus aspiration and revealed that higher ceramide C16:0 plasma concentrations were significantly correlated with larger aspirated thrombus volume, larger intracoronary thrombus burden, and poorer pre- and post-procedural thrombolysis in myocardial infarction (TIMI) flow; ceramides C24:0 and C24:1 were also associated with a larger intracoronary thrombus burden (145). Furthermore, plasma concentration of sphingomyelin, ceramide and glucosylceramide has been shown to correlate positively with high-sensitivity C-reactive protein, as a marker of inflammation in patients with myocardial infarction (146). Ceramides has also been proposed as a promising prognostic biomarker in patients with myocardial infarction. De Carvalho et al. proposed a 12-ceramide plasma signature which predicted cardiovascular death, MI, and stroke at 1 year in patients with acute MI (138). Cer (d18:0/16:0) and Cer (t18:0/12:0) and sphinganine have been shown to have high prognostic sensitivity and specificity for major adverse cardiovascular events for patients under 45 years of age with STEMI (147).

In addition, patients with STEMI have decreased plasma concentrations of S1P and sphinganine-1-phosphate, while the erythrocyte concentrations of S1P, sphingosine, sphinganine, sphinganine-1-phosphate were significantly increased on hospital admission and some of them were sustained for 2 years following infarction (148, 149). Moreover, in patients with myocardial infarction, the uninfected area of the myocardium presents a reduced S1P/Cer ratio (141). It has been shown that increased plasma concentrations of S1P correlated significantly with the occurrence of pre-infarction angina (150). Recently, Polzin et al. suggested that S1P may be responsible for the “Ang II receptor blockers (ARB)-myocardial infarction” paradox, which is a phenomenon in which ARB do not reduce the risk of myocardial infarction, despite effective blood pressure control (151). The authors revealed that patients treated for 3 months with ARB have a significantly lower plasma concentration of S1P in comparison with the patients treated with angiotensin converting enzyme inhibitors (ACEI) (151).

### Heart failure

Heart failure is a highly occurring disease, whose prevalence is considered to be 1–2% of the adult population in developed countries, and more than 10% among people over 70 years of age (152). The current terminology distinguishes three groups of patients with HF in regard to the left ventricular (LV) ejection fraction (LVEF): HF with preserved ejection fraction (EF) (HFpEF; LVEF ≥50%), HF with mildly reduced EF (HFmrEF; LVEF 41–49%), and HF with reduced EF (HFrEF; LVEF ≤40%). There are substantial differences in the pathophysiology of HFpEF and HFrEF. HFpEF is considered to derive from the compound of several risk factors and comorbidities, such as older age, female sex, obesity, hypertension, diabetes mellitus, renal dysfunction, anemia, iron deficiency, sleep disorders, and chronic obstructive pulmonary disease (153). The main underlying pathological mechanisms leading to HFpEF are impaired cardiac relaxation and/or filling, increased chamber stiffness, and higher filling pressure with ventricular pressure overload resulting from cardiomyocyte hypertrophy and impaired energetic metabolism, interstitial fibrosis, inflammation, oxidative stress, endothelial and microvascular dysfunction (153, 154). On the other hand, HFrEF is more commonly related to CAD, including myocardial infarction, valvular disease, and uncontrolled hypertension. HFrEF may result mainly from cardiomyocyte injury due to ischemic insult such as myocardial infarction, myocarditis, valvular disease with cell death due to overload and a genetic mutation. Those processes result in systolic dysfunction, extensive myocardial fibrosis, and eccentric remodeling with ventricular dilation (152–154). Sphingolipids are considered to be involved in the cellular processes which underlie the development of both HFpEF and HFrEF.

Based on the available experimental studies, it seems that in the course of HF there is an increase in the level of Cer and a decrease in S1P in the myocardium. This likely had direct implications for the metabolism and function of the failing heart.

A mouse model of post-myocardial infarction HF induced by ligation of the left coronary artery revealed increased levels of C16, C24:1, and C24 ceramides in a failing myocardium after 10 weeks following MI in comparison with the sham operated controls (155). Recently, Hoffman et al. (156) revealed that genetically engineered mice with the overexpression of cardiomyocyte-specific Krüppel-like factor 5 (KLF5; a transcription factor which regulates the metabolism of myocardial fatty acids), have increased SPTLC1 and SPTLC2 expression, and higher cardiac levels of Cer and systolic dysfunction. Based on the above data, it can be concluded that KLF5 is a transcriptional regulator of SPTLCs which contributes to *de novo* ceramide synthesis and promotes myocardial dilation in ischemic cardiomyopathy, therefore its inhibition may be a therapeutic target for ischemic HF and myocardial remodeling (156).

Nevertheless, male C57BL/6 mice with post-MI HF showed reduced myocardial levels of sphinganine and sphingosine with increased levels of S1P at a chronic phase of HF (56 days post MI). Similarly, mRNA expression of S1P1R in the LV increased 1 day post MI and then decreased 5 days post MI, while in chronic HF the expression significantly increased, which suggests that myocardial S1P/S1PR1 signaling is enhanced during the chronic phase of HF (157). It is known that HF is associated with persistently increased activity of the sympathetic nervous system and, in experimental studies, chronic administration of β-adrenoceptor agonists is an established animal model of HF. Cannavo et al. have also proven that in C57BL/6 mice with HF, ISO resulted in a S1PR1 downregulation at the level of the plasma membrane, while chronic administration of a S1PR1-selective agonist resulted in the downregulation of β1AR cardiac plasma membrane levels; both agonists significantly increased the heart-to-body weight (HW/BW) ratio (158). Whereas, long-term overexpression of S1PR1 as a result of gene therapy in chronic HF post-MI in Sprague Dawley rats resulted in significantly smaller LV internal diastolic diameter, lower LV end diastolic pressure, and higher systolic and diastolic function, which reduced the infiltration of immune cells and restored the total plasma membrane β-adrenergic receptor (βAR) density in comparison with the HF controls with physiologically lower myocardial expression of S1PR1. Therefore, the authors concluded that treatment aimed at restoring cardiomyocyte membrane expression of S1PR1 exerts beneficial effects counterbalancing the detrimental overstimulation of β1AR present in HF (158).

A few experimental studies indicate the possibility of using sphingolipids in HF. It was shown that treatment with myriocin, a SPT-inhibitor, prevented LV dilation, improved LV systolic function, reduced the extent of myocardial fibrosis and macrophage content in C57B/L6 mice with MI-induced HF (155). Moreover, in Long Evans rats with hypotensive acute HF followed by a recovery phase, exogenous administration of S1P during the recovery phase improved the heart rate, which was accompanied by the activation of the signal transducer and activator of transcription 3 (STAT3) pathway (159).

The available clinical studies confirm changes in the expression of sphingolipids in the myocardium in the course of HF. Moreover, they indicate the possibility of using some substances from the great family of sphingolipids as potential prognostic markers in patients with HF. Knapp et al. reported decreased plasma concentrations of free sphingosine and sphinganine in patients with chronic HF, both ischemic and idiopathic dilated cardiomyopathy, with no significant differences in the levels of S1P, sphinganine-1-phosphate, and ceramide in comparison with the healthy controls (160). However, later research showed that a higher plasma concentration of the C16:0/C24:0 ceramide ratio may be associated with the increased risk of HF and was associated with a lower left ventricular ejection fraction, worse global circumferential strain, higher left atrial end-systolic volume, and lower left atrial emptying fraction (161). Recently, Wittenbecher et al. analyzed the relationship of single lipid metabolites and the lipidomic networks with the risk of developing HF and highlighted that two single lipid metabolites, CER 16:0 and phosphatidylcholine 32:0, apart from several other lipidomic patterns, were significantly associated with HF risk (162). Post-mortem heart tissue samples of patients with ischemic HF showed a significant increase in sphingosine and sphinganine concentration and an almost 10-fold decrease in the concentration of S1P within the ischemic myocardium when compared with healthy controls, with no significant differences in S1PR1 expression (157). However, Pérez-Carrillo et al. (163) observed the accumulation of Cer and S1P in the HF myocardial tissue, with an increased ceramide/S1P ratio by 57% in HF hearts. They also found that 12 genes involved in sphingolipid metabolism (especially genes participating in the *de novo* and salvage pathways) were differentially expressed in HF compared with the control patients (163).

Plasma ceramide levels have been found to be increased proportionally to the New York Heart Association (NYHA) functional class and were an independent risk factor of mortality in patients with chronic HF with reduced ejection fraction (164). Whereas, in patients with ischemic HF, plasma S1P and sphingomyelin concentrations were negatively associated with LV ejection fraction and NYHA class (165). Moreover, in HFpEF patients, increased plasma concentrations of Cer 16:0 and Cer 18:0 were associated with an increased risk of death or HF admission, with no such relationship in regard to Cer 24:0 (166). Targher et al. analyzed outpatients with chronic HF irrespective of LVEF enrolled in the Gruppo Italiano per lo Studio della Sopravvivenza nell’ Insufficienza Cardiaca-Heart Failure (GISSI-HF) trial and revealed a plasma ratio of Cer(d18:1/16:0), Cer(d18:1/18:0), Cer(d18:1/20:0), Cer(d18:1/22:0),Cer(d18:1/24:1) to Cer(d18:1/24:0) were significantly related to a higher risk of cardiovascular mortality, however after adjustment for established cardiovascular risk factors, medication use, and plasma concentrations of N-terminal (NT)-pro hormone BNP (NT-proBNP), these associations were impaired (167).

### Arrhythmias and conduction disorders

Arrhythmias are a common heart dysfunction manifested as disruption of the appropriate periodicity and regularity of electromechanical activity (168). Conduction disorders, also known as heart blocks, are dysfunctions in which electrical signal production or its propagation through the myocardium is abnormal (169).

Considering the fact that sphingolipids are components of cell membranes, it can be hypothesized that sphingolipids also regulate cardiac repolarization, and therefore may play an important role in the pathophysiology of arrhythmia and conduction disorders.

The above hypothesis seems to be confirmed by experimental studies. Based on the available data, it can be assumed that particularly ceramides have the ability to regulate cardiac repolarization. Whole-cell patch clamp studies carried out on the human embryonic kidney cell line HEK293, which had been incubated for several hours with Cer, reacted with the inhibition of the human Ether-à-go-go-Related Gene (hERG) potassium channel current (a major component of rapid delayed rectifier K + current/IKr). Such an inhibition may prolong the action potential duration and QT interval, predisposing to ventricular arrhythmias and sudden cardiac death (170–172). Similarly, Huang et al. revealed that, in isolated rabbit pulmonary vein tissues, C2-ceramide regulates its electrophysiological properties, therefore it may be involved in the pathogenesis of pulmonary vein-induced arrhythmogenesis (173). Furthermore, in the rat model of lethal ventricular tachyarrhythmia (LVTA)-sudden cardiac death (SCD) (Sprague Dawley rats injected with aconitine into the tail vein), Wu et al. showed disruption of the plasma concentration of Cer, sphingomyelin, phosphatidylcholine, phosphatidylethanolamine, and phosphatidylserine after an LVTA-SCD event (174, 175). Tachycardia in Wistar rats has also been shown to change the metabolism of bioactive sphingolipids differently in each ventricle, in LV increasing the myocardial concentration of sphingosine and reducing concentrations of ceramide, S1P, and sphinganine-1-phosphate, while in RV increasing the concentration of sphingosine and sphinganine, with a reduction of S1P, sphinganine-1-phosphate, and ceramide (176).

In turn, S1P is involved in particular in the regulation of the intracellular potassium current in the myocardium. It was demonstrated that in isolated human atrial cardiomyocytes, S1P-S1PR3 signaling is responsible for the activation of the muscarinic receptor-activated inward rectifier potassium current, which has been involved in the vagally mediated regulation of the heart rate (177). Additionally, Ochi et al. (178) have proven that S1P activates a weakly inwardly rectifying K^+^ current in guinea pig atrial myocytes, which significantly shortens the action potential duration and shortens the effective refractory period (ERP). A shortening of the ERP may predispose to the development of reentry arrhythmias, such as atrial fibrillation (178). Moreover, in Sprague Dawley rats, pharmacological inhibition of S1P lyase caused bradycardia (179).

Several clinical studies appear to confirm the role of sphingolipids in the regulation of cardiac polarization. Recently, an analysis of the Cardiovascular Health Study population without a history of atrial fibrillation revealed that plasma ceramides and sphingomyelins with very long chain saturated fatty acids were associated with a reduced risk of incident atrial fibrillation, whereas ceramides and sphingomyelins with palmitic acid were associated with an increased atrial fibrillation risk (180). Moreover, in clinical settings, treatment with S1P receptor modulators was associated with side effects, such as bradycardia, atrioventricular blocks, and also probably ventricular tachycardia, which were suggested to be mainly S1PR1/S1PR3-dependent (46, 181–184). In addition, patients with atrial fibrillation had significantly lower plasma concentration of S1P in comparison with the controls with sinus rhythm, however, the exact role of S1P in atrial fibrillation needs to be further investigated (185). Furthermore, in patients with rheumatoid arthritis, psoriasis, and other inflammatory conditions who frequently had prolonged QT, administration of fingolimod prolonged the QT interval and inhibited the hERG current (186).

### Stroke

Vascular diseases of the brain are one of the most frequent causes of death and disability, and the number of cases in many countries around the world is steadily increasing. Stroke is the acute neurologic injury that occurs as a result of brain ischemia or brain hemorrhage, both of which present with different clinical manifestations and outcomes (187). Sphingolipids have been shown to play a pivotal role in the pathophysiology of ischemic stroke.

**FIGURE 5:**
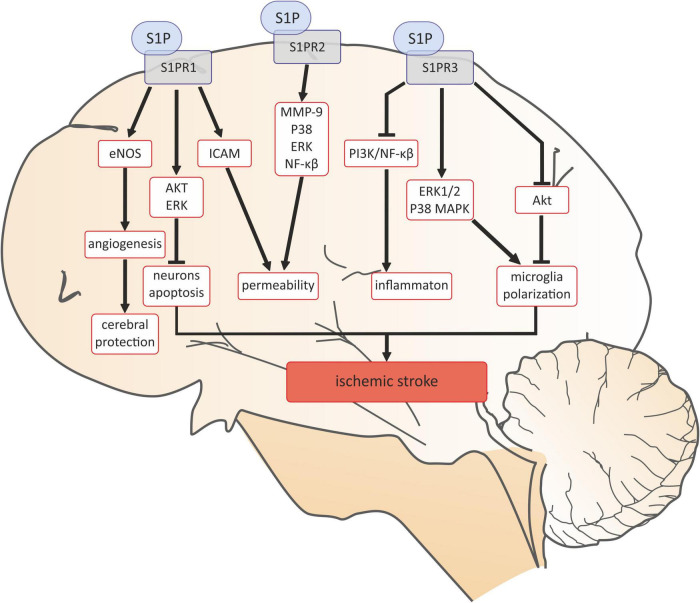
Sphingosine 1-phosphate signaling in ischemic stroke. AKT, serine/threonine protein kinase B; eNOS, endothelial nitric oxide synthase; ERK, extracellular signal-regulated kinases; ERK1/2, Ras-dependent extracellular signal-regulated kinase; ICAM-1 -intercellular adhesion molecule 1; MMP-9, matrix metalloproteinase-9; NF-κB, nuclear factor kappa-light-chain-enhancer of activated B cells; p38, p38 mitogen-activated protein kinases; PI3K, phosphoinositide 3-kinase; S1P, sphingosine-1-phosphate; S1PR1, sphingosine-1-phosphate receptor 1; S1PR2, sphingosine-1-phosphate receptor 2; S1PR3, sphingosine-1-phosphate receptor 3.

Based on the literature data, it can be concluded that sphingolipids are widely represented in the central nervous system (CNS), for example neurons and astrocytes have the ability to synthesize and release S1P (2). Moreover, expression of all the S1P receptors has been found within the central nervous system: oligodendrocytes mainly express S1PR1 and S1PR5, astrocytes: S1PR1 and S1PR3, while microglia express S1PR1-3 (2). Due to the common occurrence of S1P and its receptors in brain tissue, it can be assumed that they may also play a role in the pathophysiology central nervous system diseases, including stroke. Especially that both experimental and clinical studies suggest the important role of S1P as a potentially mediator in ischemic stroke. Ischemia increases the concentration of S1P in the brain tissue and has been suggested to be a microglial-dependent process (188–190). Exogenous administration of S1P to the healthy mouse brain activates microglia and astrocytes, inducing a local neuroinflammatory response (191). S1P may play a role in stroke through its receptors (59). Overall, all S1P receptors (SPR1-3) accelerate the development of ischemic stroke (59). It was revealed that the interaction of S1P with S1PR1 and S1PR2 play a pivotal role in the disruption of the cerebrovascular integrity thereby promoting the destruction of the blood-brain barrier (BBB) (59). Damage to the BBB throughout S1PR1 is mediated by ICAM-1, while S1PR2 may increase the activity of metalloproteinase 9 (MMP-9) and enhance expression of p38 MAPK and ERK1/2 proteins consequently activating the nuclear factor kappa-light-chain-enhancer of activated B cells (NF-kB) directly leading to an increase the permeability of cerebral vessels (192–194) (Figure 5). S1PR1, 2, and 3 have been shown to mediate the activation of microglial cells and the inflammatory response in male ICR mice, which may play an important role in the pathogenesis of ischemic stroke (192, 195). Inhibitions of S1PR1 and S1PR3 activity in the mouse model of transient middle cerebral artery occlusion/reperfusion (tMCAO) significantly reduced the brain infarct volume, improved the neurological deficit, and decreased the number of the activated microglia and expression of proinflammatory cytokines (192, 195, 196). Moreover, inhibition of S1PR2 attenuates microglial activation and microglial proliferation in the mouse post-ischemic brain after tMCAO (197). S1PR2 has also been found to play a pivotal role in the disruption of the cerebrovascular integrity in the experimental model of stroke (198). The role of S1PR3 in promoting macrophage proliferation is perhaps the best explained. It has been reported that the mechanisms of action of S1PR3 were associated with ERK1/2 and p38 MAPK activation and with Akt inactivation (195) (Figure 5). However, it has been shown that the interaction of S1P with S1PR1 can also protect the brain after ischemic stroke by inhibiting the inflammatory response and neuronal apoptosis, as well as promoting angiogenesis (59) (Figure 5).

In addition, the activity of enzymes leading to the formation of S1P, i.e., SPHK1 and SPHK2 play a role in the ischemia-induced brain injury, however, their activity is opposite. *In vitro* studies have revealed that neurons subjected to glucose-oxygen deprivation have an increased expression of SPHK1 and pro-inflammatory mediators in the primary microglia, while the inhibition of SPHK1 attenuates the microglial induction of the proinflammatory mediators by ischemic neurons (199, 200). Zheng et al. in a mouse model of stroke induced by middle cerebral artery occlusion (MCAO) noticed an increase in the expression of SPHK1 predominantly in the microglia, and a reduction in the expression of inflammatory mediators in the cortical penumbra (199). Additionally, pharmacological inhibition of SPHK1in the same animal model decreased the size of infarction, preserves the integrity of the blood-brain barrier, and improves the neurological deficits after reperfusion (199, 201). Su et al. have shown that SPHK1/S1P mediates neuroinflammation and neuronal apoptosis *via* tumor necrosis factor receptor-associated factor 2 (TRAF2) and NF-κB in the activated microglia in the rat model of cerebral I/R (202). Furthermore, inhibition of SPHK1 by siRNA reduced the production of interleukin-17A (IL-17A) and decreased the degree of microglia subjected to oxygen-glucose deprivation, suggesting that SPHK1/S1P mediates the expression of IL-17A in the activated microglia, thereby inducing neuronal apoptosis in cerebral I/R (200). On the other hand, SPHK2 seems to exert beneficial neuroprotective effects in cerebral ischemia. Yung et al. have shown that C57BL/J mice exposed to pre-conditioning with isoflurane or hypoxia before tMCAO have upregulated cerebral SPHK2 and the protective effects of pre-conditioning, including reduced infarct volumes and improved neurological outcomes are attenuated in animals lacking SPHK2 (SPK2^–/–^) (203). Wacker et al. also showed that SPHK2 inhibition by dimethylsphingosine in Swiss-Webster ND4 mice, subjected to hypoxic pre-conditioning and tMCAO, reduced infarct volume, neurological deficits, and ipsilateral edema (204). Moreover, in mice subjected to tMCAO, the knockout of SPHK2, but not SPHK1, increased the ischemic lesion size and worsened the neurological function (205).

In addition, tMCAO, as well as lethal ischemia, increased brain tissue Cer concentration in rodents (206, 207). Yu et al. (208) reported that in wildtype mice tMCAO increases the activity of acid sphingomyelinase, increases the ischemic cortical tissue concentration of Cer, and increases the production of reactive oxygen species, which was absent in mice lacking acid sphingomyelinase. Moreover, mice lacking acid sphingomyelinase had a decreased degree of apoptosis and their neurological deficits were improved in comparison with wildtype animals (208). In the rat model of chronic cerebral ischemia induced by the clipping of the common carotid arteries, Ohtani et al. revealed that after 14 days there is a significant increase in brain tissue activity of acid sphingomyelinase and Cer concentration, specifically in astroglia (209).

Clinical trials confirm the important role of sphingolipids in the pathogenesis of stroke. Namely, the Cer signaling pathway appears to exert a detrimental effect in the pathophysiology of stroke. *In vitro* studies on the human neuroblastoma cell line SHEP subjected to experimental ischemia (serum/oxygen deprivation) have increased the activity of acid sphingomyelinase and ceramide production, which reached a maximum of 30 min following reoxygenation (206). Moreover, in patients after acute ischemic stroke, Lee et al. (210) have proven that the plasma concentration of S1P and very long chain ceramides are significantly lower in comparison with the non-stroke controls. A group of patients who suffered from ischemic stroke also had higher concentrations of long chain ceramides. Moreover, greater levels of Cer(d18:1/18:0), Cer(d18:1/20:0), and Cer(d18:1/22:0) measured at 48–72 h following the onset of stroke were predictors of poorer functional outcomes (210). Gui et al. (211) have shown that plasma levels of Cer(d18:1/16:0), Cer(d18:1/22:0), and Cer(d18:1/24:0) were significantly higher in stroke patients than in patients from the control group. Higher plasma levels of C16:0, C22:0, and C24:0 were associated with the clinical severity of stroke, and patients with minor stroke (defined on the National Institutes of Health Stroke Scale (NIHSS) <6) had lower ceramide serum levels that those with moderate-to-high clinical severity (211). Fiedorowicz et al. found two sphingolipid ratios (Sph-1-P/Cer-C24:1and Cer-C24:0/Cer-C24:1) to be strongly characteristic and both had diagnostic potential in ischemic stroke (212). Furthermore, patients with post-stroke depression (PSD) have significantly higher plasma concentrations of Cer16:0, Cer18:0, Cer24:0, and Cer24:1 in comparison with non-post-stroke depression (non-PSD) patients. Moreover, the specific ceramides C16:0 (vs. major depression; MD), C18:0 (vs. MD), and C16:0 (vs. Non-PSD, *P* = 0.002) can be used as predictive risk factors for diagnosis (213). Similar results were shown by an open-label, evaluator-blinded, parallel-group clinical pilot trial, where patients, with an anterior cerebral circulation occlusion and onset of stroke that exceeded 4.5 h and who had received standard management with additional fingolimod orally for3 consecutive days, had lower circulating lymphocyte counts, milder neurological deficits, better recovery of neurological functions, and less enlargement of lesion size between baseline and day 7 (214), which indicates the possibility of using sphingolipids in the pharmacotherapy of strokes in the future.

## Conclusion

Sphingolipids are not only an integral part of the cell membrane, but also play an important role in the pathophysiology of cardiovascular diseases, such as hypertension, coronary artery disease, heart failure, arrhythmias, and stroke. Moreover, recent experimental and clinical studies indicate the possibility of using sphingolipids in the pharmacotherapy of the above diseases. However, more research is needed to better understand the role of sphingolipids in cardiovascular disease.

## Author contributions

SB-J, AC-J, and KC: conceptualization. SB-J, PJ, WŁ, and KC: literature review. SB-J, PJ, WŁ, AC-J, and KC: writing—original draft preparation and writing—review and editing. KC and AC-J: supervision. KC: project administration. All authors have read and agreed to the published version of the manuscript.
